# A precipitation gradient drives change in macroinvertebrate composition and interactions within bromeliads

**DOI:** 10.1371/journal.pone.0200179

**Published:** 2018-11-28

**Authors:** Laura Melissa Guzman, Bram Vanschoenwinkel, Vinicius F. Farjalla, Anita Poon, Diane S. Srivastava

**Affiliations:** 1 Department of Zoology, University of British Columbia, Vancouver, British Columbia, Canada; 2 Community Ecology Laboratory, Vrije Universiteit Brussel, Ixelles, Belgium; 3 Department of Ecology, Universidade Federal do Rio de Janeiro, Rio de Janeiro, Brazil; Uniiversity of Padova, ITALY

## Abstract

Ecological communities change across spatial and environmental gradients due to (i) changes in species composition, (ii) changes in the frequency or strength of interactions or (iii) changes in the presence of the interactions. Here we use the communities of aquatic invertebrates inhabiting clusters of bromeliad phytotelms along the Brazilian coast as a model system for examining variation in multi-trophic communities. We first document the variation in the species pools of sites across a geographical climate gradient. Using the same sites, we also explored the geographic variation in species interaction strength using a Markov network approach. We found that community composition differed along a gradient of water volume within bromeliads due to the spatial turnover of some species. From the Markov network analysis, we found that the interactions of certain predators differed due to differences in bromeliad water volume. Overall, this study illustrates how a multi-trophic community can change across an environmental gradient through changes in both species and their interactions.

## Introduction

Ecological communities can change across spatial and environmental gradients in three main ways: the composition of species can change, the strength of interactions between species can change, or the presence of the interactions can change [1,2]. Species composition can vary across an environmental gradient if the environment filters particular traits [3,4], and across space if species differ in their dispersal abilities [3,4]. Even when species are found together across a gradient, the presence or strength of interactions between these species can vary between sites on the gradient. For example, consumers may find prey more efficiently in structurally simple habitats, resulting in stronger interactions than in complex habitats [5]. Consumption rates can also be higher in warmer sites, due to temperature-dependence of metabolic rates [6]. Here we combine multiple analyses to show how environmental and spatial gradients affect both the composition and interactions of species in a multi-trophic ecological community.

Estimating changes in species interaction strengths between sites is notoriously difficult, much more so than estimating changes in community composition (e.g. [7,8]). For example, pairwise competition experiments consider interactions between two species. These experiments would need to be performed at multiple environments to estimate changes in interaction strengths [9] and they ignore the influence of other species in the estimates of interaction strengths. In order to measure interactions in a community context (i.e. including indirect effects), researchers have experimentally removed one species from the system and assessed the impact on the whole community. However, this approach cannot reconstruct the strength of the interactions between all members of a community, only the interactions between the removed species and the rest of the community [10].

Inferring species interactions from observational data, as opposed to experimental manipulations, has the advantage of observing the end result of multiple direct and indirect interactions. For instance, combining observations of prey abundance and predator foraging rates can provide information on interaction strengths [11]. However, this method cannot estimate indirect interactions. Another popular method, checkerboard analyses, can determine if observational patterns in species co-occurrence differ from random assembly[12,13]; that is, checkerboard analyses attempt to estimate the effect of competition in shaping the distribution of species. However, such analyses do not explicitly test for differences in interaction strengths, nor account for indirect interactions between species.

Markov networks are a promising method to get information about species interaction strengths from observational data while controlling for indirect interactions between species [14]. A Markov network relates the probability of the occurrence of multiple species at a site to two parameters: α and β. α determines how much the presence of a given species contributes to the probability of observing the presence and absences of all species in that site. β determines how much the co-occurence of a given pair of species contributes to the same probability. Given an observed vector of presences and absences, we can use maximum likelihood estimation to obtain the parameters α and β [14]. If species are less likely to occur together, their interaction strength (parameter β) will be negative. And, conversely, if species are more likely to occur together, their interaction strength (parameter β) will be positive. This method was developed for competitive communities that show a checkerboard distribution. A checkerboard distribution refers to an arrangement where two species are found to always occupy different patches. This distribution might be the outcome of some exclusion process (competition or predation) [12]. This reasoning suggests that we can infer interaction strengths in certain types of simple multi-trophic communities that also display checkerboard distributions (see also [14]). Although a predator cannot persist in the absence of its prey in a closed system, open systems with a high colonization rate of the prey and a high predation rate can also display a checkerboard distribution between the predator and the prey. When the predator consumes its prey to extinction, we may find the predator on its own. If the prey has a high colonization rate, it can colonize patches where the predator is absent. These colonization—extinction dynamics can lead to a system with patch dynamics (e.g. [15]). In other words, the spatial scale can affect the degree of co-occurrence observed between predators and prey; at small scales, effective predators should reduce or eliminate their prey (negative co-occurrence) while at larger scales predators and prey should positively co-occur [16]. Here we define interaction strength as a measure of the degree of co-occurrence between pairs of species, akin to measuring the correlation between the occurrence of two species [17]. Note that this definition of interaction strength does not map to biomass or energy flux between trophic levels, but rather conforms to one of Berlow et al.’s [17] definitions of interaction strengths as a statistical pattern of co-occurrence at a given spatial scale.

**Fig 1:**
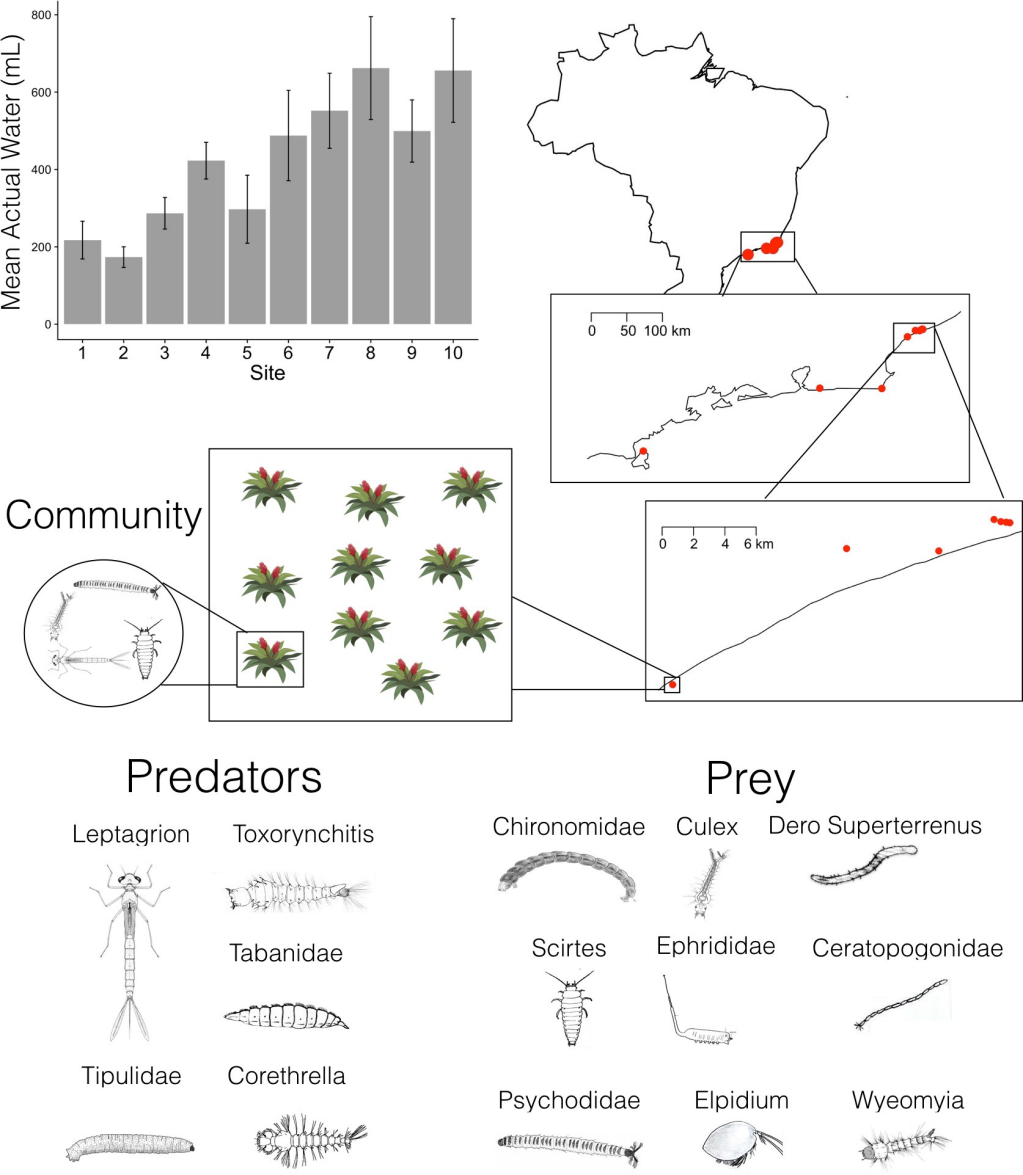
Sites are located along the eastern coast of Brazil. A site is comprised of ten bromeliads found within 100 meters of each other. Ten sites were sampled, with a hierarchy of distances between bromeliads (nested boxes, right side of diagram). The mean actual water found in the bromeliads from each site is shown. Bars represent mean and standard error of the mean. We estimated the standard error by dividing the standard deviation by the square root of the number of observations. Sites 1 to 10 are ordered from north to south. A community is the set of species found in one bromeliad. The bromeliad macroinvertebrate community is comprised of predators, mesopredators and prey.

Despite their potential, Markov methods have thus far not been used to reconstruct interactions in real food webs along environmental and spatial gradients. Good candidate ecosystems for such analyses are insular systems with simple food webs that occur over wide geographic areas. In such ecosystems, species interactions are contained within each replicate of the system and the environment can vary between systems. A classical food web model system that fits these criteria are the aquatic communities that live inside bromeliad plants in the Neotropics; these communities often occur as clusters that exchange species via dispersal. Bromeliad plants have leaves that interlock, forming a cavity where water accumulates. Inside these cavities, communities of aquatic invertebrates form a food web (Fig 1). In these communities, a suite of voracious predators can limit the abundance of prey species [18], and prey colonization is rapid [19]. In addition, multiple studies have shown that environmental variation (such as water volume) can determine the presence of certain species and mediate the interactions between some predators and their prey [20,21]. Thus bromeliad communities are a suitable model system to test how environment and space can affect species interactions and community composition [22,23].

Making use of this model system, we explored three main questions. First, we tested whether environmental conditions varied between our sampling sites, located along a geographic gradient. Due to spatial variation in precipitation at the time of sampling, we would expect that sites will vary in the amount of water present in the plants. Second, we describe change in community composition along this geographic gradient, and then partitioned this between-site variance (i.e. beta diversity) into either spatial turnover of species or nestedness of species assemblages—specifically nestedness of community composition as a geographic pattern. We expect that beta diversity would be driven mostly by nestedness of species assemblages. Specifically, since the amount of water in the bromeliads determines habitat size, we expect that lower water volumes reduce diversity in the community, and that sites with lower water volumes would have a subset of the species of the sites with higher water volumes [24,25]. Third, we used Markov networks to quantify species interactions at each site. We explored whether difference between sites in the strength of species interactions could be explained by geographic variation in environmental conditions. We expect that species interactions would vary along this gradient, since water volumes determine the ability of some predators to persist, and the amount of habitat available for catching prey.

## Methods

### Model system

Tank bromeliads accumulate water inside their leaf axils, providing habitat for communities of aquatic macroinvertebrates [26]. Inside each bromeliad, these aquatic macroinvertebrates interact to form a food web comprised of detritivores, filter feeders, intermediate predators and top predators. Bromeliad macroinvertebrate communities are known to be particularly sensitive to changes in precipitation, since this can change the amount of habitat available for the invertebrates [27]. For example, drought in bromeliads is known to reduce growth rates of some invertebrate species [21]. Therefore, we expect that changes in precipitation have the potential to substantially affect species interactions and community composition.

### Study area

The study area was located in the sand dunes of coastal Brazil (Fig 1), in the states of Rio de Janeiro and São Paulo. We sampled ten sites, seven of which were within the Jurubatiba National Park in Rio de Janeiro state, Brazil. The other three sites were located in the sand dunes of Arraial do Cabo (Rio de Janeiro), Marica (Rio de Janeiro), and Ilha Bela (Sao Paulo). This sampling design resulted in the sites closest to Jurutabita National Park receiving low precipitation, the sites close to Marica receiving intermediate precipitation, and the sites closest to Ilha Bela and Arraial do Cabo receiving high precipitation in the month immediately before sampling (February and March 2015, Figs A and B in S2 File). Permit number 47164–1 was provided by Ministério do Meio Ambiente (MMA), Instituto Chico Mendes de Conservação da Biodiversidade (ICMBio) and Sistema de Autorização e Informação em Biodiversidade (SISBIO). This field study did not involve endangered or protected species.

### Sampling

We sampled all macroinvertebrate communities between March and May 2015. In each site, we dissected ten bromeliads (totalling 100 bromeliads across all sites) to collect all the invertebrates in each plant. The invertebrate samples were preserved in 99% ethanol. Macroinvertebrates were counted and identified to genus level whenever possible. Overall we identified between 11 and 16 genera for each site. For every bromeliad, we measured a suite of environmental variables to assess the amount and quality of habitat available to the invertebrates (S2 File).

### Data analysis

#### Environmental variation between sites

In order to test whether sites did indeed vary in environmental conditions, we performed an ANOVA for most of the environmental variables. For two variables measured on a percentage scale, oxygen saturation and canopy cover, this ANOVA procedure was inappropriate so we used an analogous generalized linear model specifying a binomial family error distribution (Table A in S2 File).

#### Compositional variation between sites

**Fig 2:**
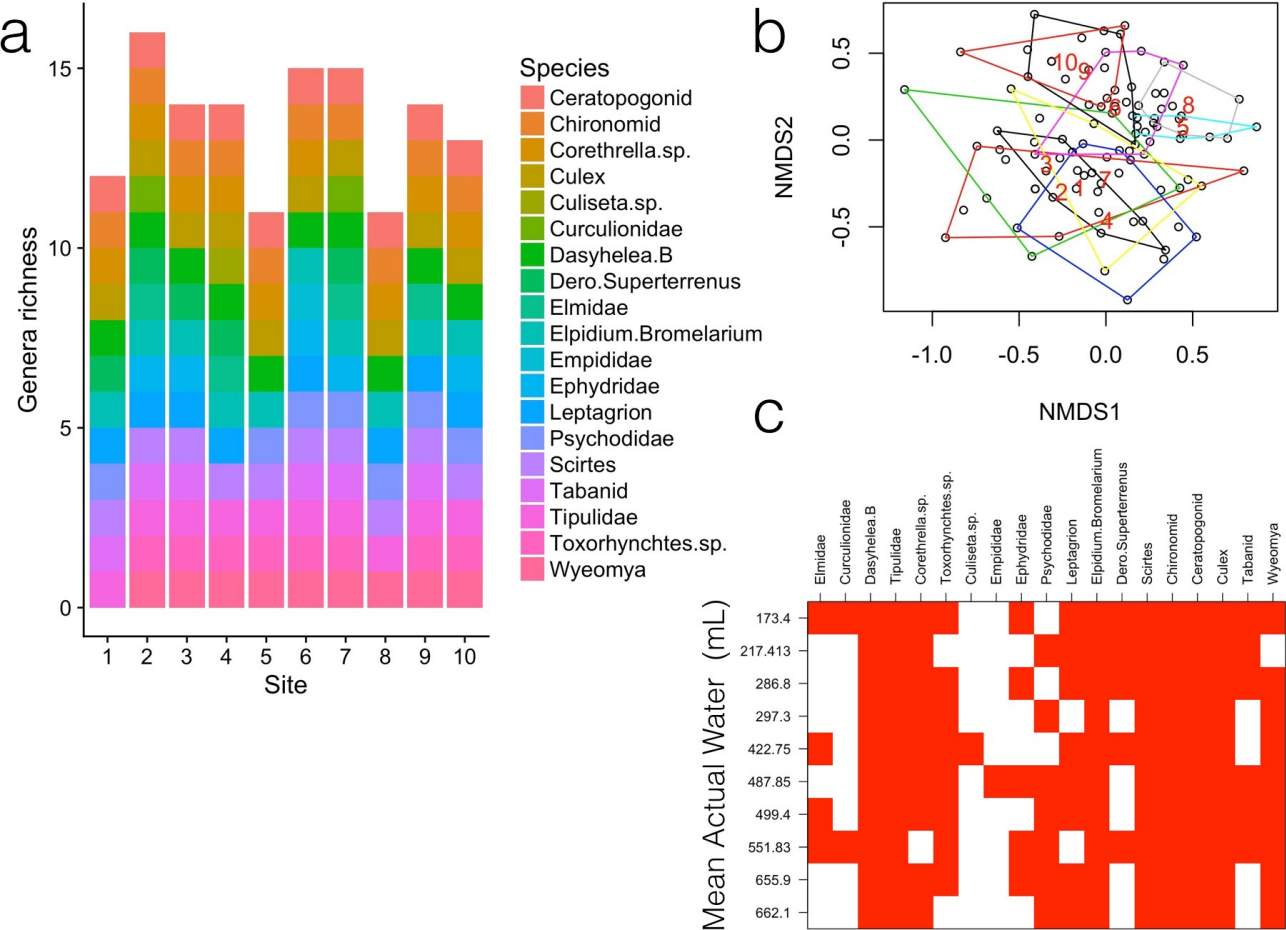
Community composition across sites. a) Richness is relatively constant between sites. b) The community composition of every bromeliad is compressed into two axes. Each site is represented by a polygon containing all bromeliads within that site. The polygons with higher overlap suggest that those sites have more similar community composition. The area of the polygons represents the differences in community composition within a site (beta diversity). While most of the polygons have a relatively similar area (resulting only in a marginal different in community dispersion), we find that there are three main clusters of overlap between the sites (Sites 1, 2, 3, 4 and 7 overlap and then sites 5 and 8, and 10 and 9 overlap). The stress of the NMDS (non-metric multidimensional scaling) plot was 0.26, consistent with a good, but not great, representation of the communities in two dimensions. c). A genera (columns) by sites (rows) matrix with sites ordered based on mean actual water, where sites with low water volumes are at the top of the graph and sites with high water volumes are at the bottom of the graph. If differences in beta diversity occur mainly through nestedness, the sites at the top would have emptier communities (more white) than the sites at the bottom. Beta diversity is mostly due to species turnover and not nested loss of species along the gradient.

We tested for differences in community composition between sites using permutational multivariate analysis of variance using distance matrices (function *adonis* in R package *vegan*, hereafter referred to as Adonis). Multivariate tests of dispersion (function *betadisper* in R package *vegan*) were used to test for differences in community variation (beta diversity) between geographic sites. We summarized abundances according to genus so that these results would be comparable with the species interactions analyses. For the *Adonis* analysis, we tested if bromeliads from different sites and containing different water volumes differed in community composition. We used water volume in this analysis since, of all the environmental variables, it differed the most between sites (Table A in S2 File). For the multivariate test of dispersion, we tested if bromeliads from different sites differed in their beta diversity, where within-site beta diversity was measured as the average dissimilarity of bromeliad invertebrate communities from the centroid in multivariate space [7,28]. To visualize the differences in community composition and dispersion, we used non-metric multidimensional scaling (NMDS) plots [7] (Fig 2B). An NMDS plot shows both the differences between sites in their average community composition (position of centroids) as well as differences between sites in beta diversity (the standardised residuals around the centroids).

To further understand our results, we partitioned beta diversity between two patterns: nestedness of assemblages and spatial turnover of species. Nestedness of species assemblages occurs when some sites have a smaller subset of the species from other richer sites [24]. This pattern could result if lower water volumes in bromeliads exclude certain species without replacement. Spatial turnover of species occurs when some species are replaced by others [24]. This pattern could result if some species can persist in low water volumes and other sets of species can persist in high water volumes. To calculate the different portions of beta diversity we used Baselga’s method [24], where Sørensen dissimilarity (β_SOR_) is partitioned into pure spatial turnover (β_SIM_) and nestedness (β_NES_) (See S1 File for details in the equations used). β_NES_ is not an absolute measure of nestedness but instead a measure of the dissimilarity of communities due to the effect of nestedness patterns. To visualize nestedness and turnover we used a nestedness and degree fill plot (Fig 2C).

Overall, we tested differences in community composition between sites using Adonis, compared the differences in beta diversity between sites using Betadisper, and finally evaluated if the differences in community composition between sites can be attributed to species nestedness or turnover using partitioning of beta diversity. Adonis tests if the differences in community composition between sites are significant, and partitioning of beta diversity relates patterns in either nestedness or turnover to the compositional dissimilarity between sites.

#### Species interactions

To obtain species interactions strengths, we used Markov network analysis [14]. This method does not make any assumptions about the topology of the food web, nor do we have to define which species might interact with each other. The method calculates the conditional species interaction strength given the presence/absence data using maximum likelihood estimation. We summarized abundances according to genus, to reduce computational complexity. The trophic role of bromeliad aquatic invertebrates is highly conserved at the genus level [29], so we likely have not averaged over different trophic interactions with this approximation. The abundance data of each genus were transformed into presence/absence data. We performed Markov Network analysis separately for each site [14]. The output of this analysis is the relative interaction strength for every pair of species in the site. We used a logistic density function for the prior distribution of interaction strengths; after running the model the final distribution of interaction strengths tended to be normal with a mean close to zero. We performed two validations for the Markov Network analysis (S3 File).

#### Methodological limitations of Markov networks

The biggest advantage of the Markov network approach is that it considers indirect interactions such as intraguild predation, which commonly occurs in container habitat food webs [30,31]. Even though Markov network analyses only calculate symmetrical interaction strengths, we argue that it is suitable for analysing trophic asymmetrical interactions when other information, such as natural history, is available [14]. Markov network analyses were designed for competitive interactions, however, competitive interactions are also known to be asymmetrical [19,32]. Despite species interactions being asymmetrical, one direction of the asymmetry is often stronger than the other. For example, in top down control, the negative effect of the predator in depressing prey abundance is often much stronger than the positive effect of the prey in supporting predators, and therefore the overall interaction is negative. Another shortcoming of this method is that, if a species is very rare at the regional scale due to dispersal limitation or habitat filtering, it may appear to be negatively interacting with many species. To reduce this problem, we only used species that were present in at least two bromeliads and the analysis was done at the site scale where most bromeliads experience the same climatic conditions. Furthermore, since bromeliad invertebrates prefer particular bromeliad sizes [33], we chose the same broad range of bromeliad sizes for every site, to ensure that we obtained the spectrum of species present in the site. After ensuring that the interpretations of the model were consistent with the natural history known of the system (i.e. the Markov network analyses correctly identified the trophic interactions of known predators and prey), we were able to check if species had different types of interactions over an environmental gradient.

#### Effect of environment on species interactions

Once we were able to confirm that Markov Network analysis correctly distinguished between predators and prey in terms of the predominant sign of interactions, we could then examine if the environment explained differences between sites in the relative strength of either positive or negative interactions. For this analysis, we separated negative from positive interactions to assess how interaction strength (within a particular sign) changes with the environmental variables, based on linear regression. We also used quantile regression to assess how interaction strengths (positive and negative) change with the environmental variables. Quantile regressions are useful when there is unequal variation in the data and therefore there might be more than one slope describing the relationship between response variable and predictor. Quantile regression is also more robust to outliers than mean regressions [34]. The linear regression and quantile regression p-values were adjusted using the Holm correction for multiple comparisons. To confirm the robustness of our results, we performed a permutation analysis by shuffling community composition (Figs F-H, Table E in S4 File).

For the species interaction analyses we used the *rosalia* package [35], all multivariate analyses were performed using the *vegan* package [36], mixed effect models were performed using *lme4 [37]* and *car [38]*, and all analyses were done using the R programming language [39].

## Results

### Environmental variation between sites

The only two environmental variables that significantly differed between sites were maximum and actual water volume in bromeliads (Table A in S2 File), and of these two, the most pronounced gradient was observed in the actual water volume in the bromeliads (F_10, 90_ = -3.854, P = 0.0003, Fig 1). We therefore focus on actual water volume as the major environmental gradient for the remainder of the analyses.

### Community variation along an environmental gradient

Community composition differed between sites, depending on the actual water volume in the bromeliads (F_9, 90_ = 4.649, P = 0.001, Fig 2A and 2B). However, beta diversity, measured as multivariate dispersion in composition around site centroids, differed only marginally among sites (F_9, 90_ = 1.966, P = 0.052). These site differences in beta diversity were mainly driven by the sites that were the furthest apart geographically and differed the most in the actual water contained in bromeliads (Table C in S4 File). The difference in community composition between sites was mostly due to species turnover (70%) and not due to nestedness (30%, Fig 2C). Therefore, contrary to our initial predictions, species were not progressively lost along the gradient of actual water in the bromeliads and species richness per bromeliad was relatively constant across the gradient (Fig 2A).

### Effect of environment on species interactions

**Fig 3:**
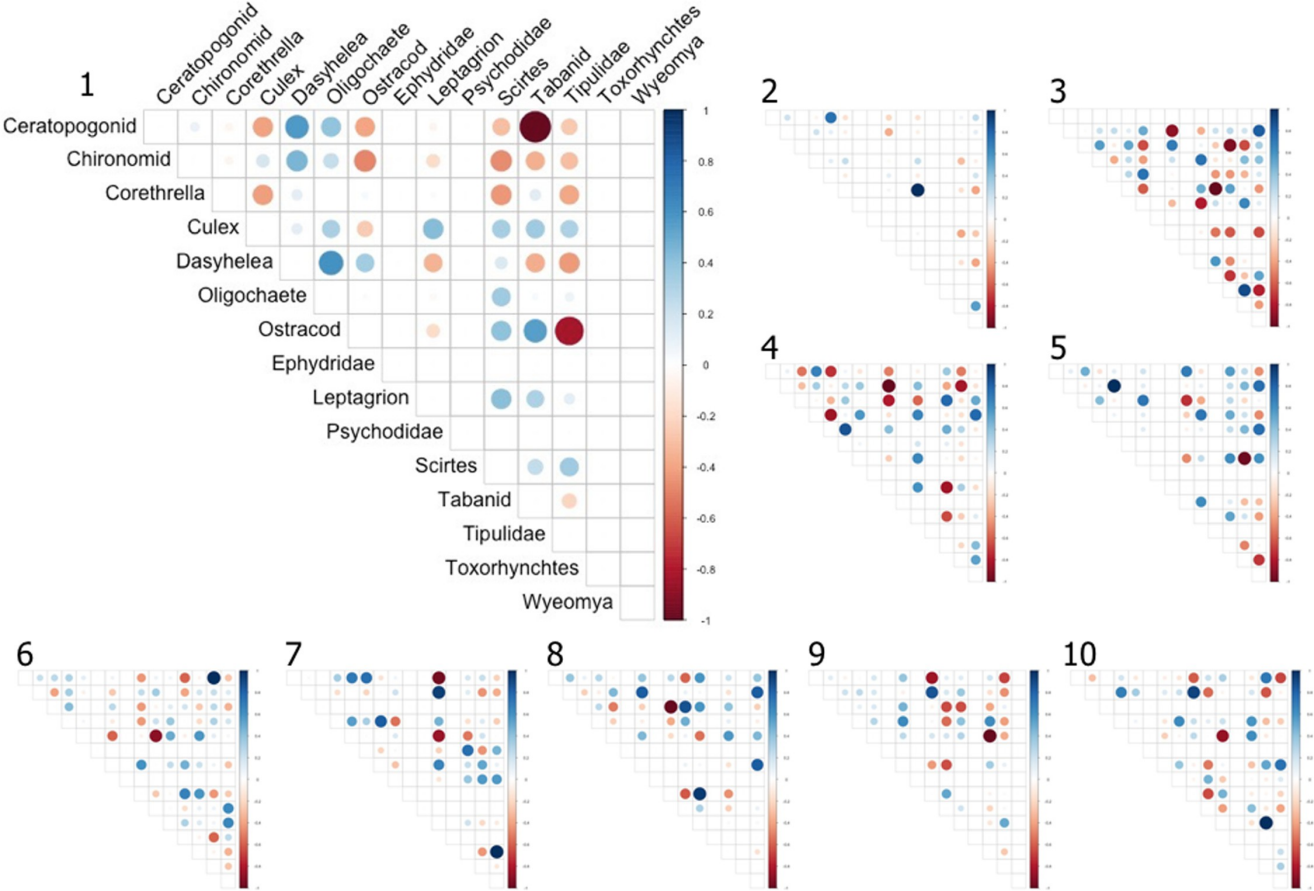
Relative strength of species interactions in every site. Species interactions are scaled to 1. Where rows or columns are empty, that particular species is not in that site. Blue indicates positive interactions and red indicate negative interactions. Positive interactions represent species than tend to co-occur, negative interactions represent species that do not tend to co-occur.

**Fig 4:**
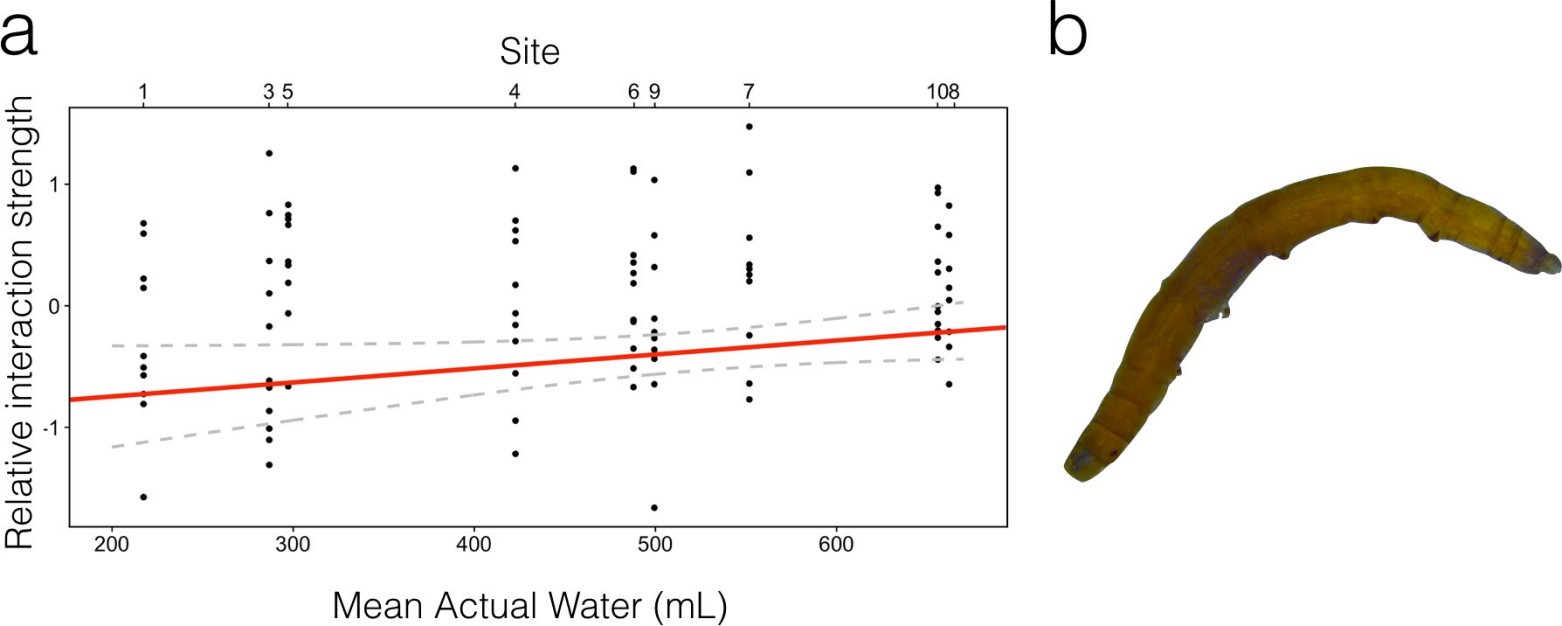
a) The tipulid has more negative interactions at low water volumes (Intercept = -0.98, se = 0.27, slope = 0.001, se = 0.0004). The red line represents the first quantile regression. Tipulids are absent from site 2, so this site is absent from this regression. Dashed lines represent predicted confidence intervals. b). Image of a Tipulid larvae.

As the majority of genera were found in most sites, we could ask how each genus differed across the large scale environmental gradient in terms of interaction type (i.e. sign) and strength (i.e. magnitude) with other community members. For every pair of genus we obtained an interaction strength in every site (Fig 3). Using site means of actual water as the environmental gradient, we found that the relative strength of positive and negative interactions remain constant between sites for most genera, but for *Tipulidae*, *Wyeomyia* and *Elpidium* the relative strength of negative interactions diminished with site water volume (Fig E, Table D in S4 File). That is, sites whose bromeliads contained less water on average tended to have stronger negative interactions community members and either *Tipulidae* (linear regression: β = 1.179 x 10^−3^, P value = 0.017), *Wyeomyia* (β = 8.254 x 10^−4^, P value = 0.062) or *Elpidium bromeliarum* (β = 1.257 x 10^−3^, P value = 0.016). Arguably, quantile regression might be better suited to detecting changes in the distribution of interactions, in which case only the *Tipulidae* interactions are still related to the mean water volume, even after the results were adjusted for multiple comparisons (first quantile regression: β = 1.151 x 10^−3^, P value = 0.05, Fig 4A).

Even though difference between sites in interactions strengths could be due either to changes in the per capita interaction strengths between specific taxa, or changes in the pool of species available for interactions, we find that low volume sites do not progressively lose species (i.e. there is no nested loss of species, Fig 2C). We also found that the absence of a species due to low water volume was not related to the interaction strength between those species and the tipulid (Fig I in S4 File)

## Discussion

The main conclusions of this study were threefold. First, we found that, due to variation in precipitation, the actual water contained in the bromeliads was the main variable that consistently varied between sites. Second, we found that sites differed in both the average composition within bromeliads and the difference between bromeliads in composition (beta diversity). However, most of the effect of sites on beta diversity was due to the turnover of some species and not due to sequential loss of species being filtered by the environmental gradient (i.e. between-site turnover vs. between-site nestedness). Third, after extensively validating a Markov network approach for trophic interactions, we found that interactions between tipulids and other species changed along a site gradient in actual water volume; sites with lower water volume had more intense negative interactions.

Our sites were located along a precipitation gradient with those in the south-west of our gradient receiving less rainfall than those towards the north-east (Figs A and C in S2 File). This gradient was reflected in the amount of actual water found in the bromeliads (Fig C in S2 File), but not other aspects of the bromeliad environment (e.g. water chemistry). Low water volumes can affect bromeliad communities through a multitude of mechanisms: (i) low water volumes can select species whose traits allow them to be tolerant to drought [40]; (ii) low water volumes decrease habitat size thereby decreasing the size of the community [41] (iii) high water volumes allow higher trophic levels to persist [21].

Overall, community composition differed along the geographic gradient in bromeliad water volume. However, this difference is not driven by the sequential loss of species along this gradient, but instead turnover in species identity. For example, the oligochaete *Dero superterrenus* was more common in the drier sites, and *Polypedilum* chironomids in the wetter sites. Such turnover may be related to the life history of organisms: oligochaetes reproduce within bromeliads, and so are resident year round, whereas larval chironomids require terrestrial adults to oviposit eggs and adults may delay oviposition until most bromeliads in the site are water-filled. Previous studies have shown that the functional traits of bromeliad invertebrates determine their response to water levels within bromeliads: taxa able to survive low water conditions are characterized by small size and deposit or filter feeding whereas taxa able to rapidly colonize full bromeliads are characterized by drought-tolerant eggs and short generation times [40]. Since species traits determine their response to altered environmental conditions, selection of species through their traits can alter not only the size of a community but also its structure [1]. More generally, if species differ in their optimal environment due to their life history and tolerance traits, we would expect that the arrangement of sites along an environmental gradient would cause a turnover in species composition due to species sorting mechanisms or when early successional species are gradually lost [42,43]. Note that this species turnover occurs despite constant species richness between sites, regardless of water volume. However, within site, species richness increases with bromeliad water volume, as shown in previous studies of bromeliad invertebrates [44].

Even though Markov network analysis can be used in food webs, the low number of degrees of freedom in compositional data only allow us to estimate one interaction value per species pair [14]. However, we can use information from the natural history of the system to allow us to interpret these interactions [14,16]. We looked at the type of interactions that prey and predators participated in, knowing prior to the analysis which species were predators and which species were prey. We found that the top predator *Leptagrion andromache* dominates negative interactions, as expected from a generalist predator known to have high per capita impact on its prey [19]. Furthermore, we found that predatory species were more likely to participate in net negative interactions and prey species were more likely to participate in net positive interactions, confirming that the Markov network approach could detect trophic interactions. This however, does not mean that all predator-prey relationships are necessarily detected via negative interaction strengths.

Our Markov analyses indicated that, while overall species interactions are similar in sign and strength along the water volume gradient, for three genera there is a consistent pattern of strengthening negative interactions in sites with lower water level. This pattern was particularly robust for tipulids. There are two possible mechanisms for this result. First, species that have only weak interactions with the three genera may become absent at sites with low water volumes, allowing stronger negative interactions to influence the mean. If this mechanism was operating, then the pattern should disappear with quantile regression. Indeed for two genera it does, but not for tipulids. The second mechanism is that many of the negative interactions intensify in strength as site water volumes diminish. This mechanism is consistent with the patterns seen in the tipulids.

Tipulids may show stronger negative interactions at low water volumes because they become generalist predators. This mechanism is supported by previous research, which found that tipulids in Costa Rican bromeliads supplement detritivory with opportunistic predation under drought [20]. These researchers hypothesized that decreasing water volume in a bromeliad restricts the space for prey movement, and therefore the tipulids can become more effective predators. Other manipulative experiments confirm that bromeliad predators are more effective in smaller water volumes [45]. Generally, from a biomechanical perspective, consumption rates of predators should depend on habitat dimensionality because it influences the cost of locomotion and the probability of prey escape [46].

Tipulids thus appear to be facultative predators, opportunistically switching from detritivory to predation. Facultative predators feed both on plant matter and animals at the same developmental stage; they represent a case of non-obligate omnivory [47]. Facultative predation may constitute an adaptive strategy in habitats with high variability of food sources, and allow species to withstand changing environments [33,47]. Bromeliad habitats are known to be very variable, with water levels that fluctuate year around [48]. Therefore facultative predation may be a favourable strategy in these systems.

Our study adds to the evidence that trophic interactions may change with climate in bromeliad infauna. Over a much larger geographical gradient, Romero et al. [49] found that cooler, less seasonal climatic conditions resulted in stronger top-down control from predators, based on biomass ratios of top predators to detritivores as a proxy for interaction strength. In this study, Romero et al. focused on odonate larvae as top predators. Here, we find that tipulid predation intensified in warmer, more seasonal sites. An intriguing topic for future study is whether seasonal droughts in Rio de Janeiro state, Brazil, shift predation from odonates to tipulids.

Our study reinforces the general point that ecological communities can change along an environmental gradient through three main ways (i) through the turnover of species, (ii) through the change in species interactions, or (iii) through the presence or absence of interactions [1]. Here we found the first two mechanisms are contributing to the changes in community composition along a gradient of water within bromeliads.

## Conclusion

In this study we provided evidence for changes in community structure along an environmental gradient through two mechanisms. First we showed that community composition differed along a gradient of actual water in macroinvertebrate communities due to the turnover of some species. Second we showed that species interactions also differed along this gradient. In our system, lower water levels likely changed the effectiveness of different predation strategies reflected in different more negative species interactions. The notion that the same actors might be active in a totally different play implies that it may not be recommended to directly link species composition to ecosystem functioning, as attempted in many recent studies [50,51]. Broader applications of the Markov approach to assess interaction strengths could assist studies that aim to explain differences in functional aspects of ecosystems that cannot be attributed to differences in species composition.

## Supporting information

S1 File Partitioning beta diversity. We partitioned beta diversity using Baselga’s (2009) method. In this supplementary file we show the equations used to partition beta diversity.
(PDF)

S2 File Environmental variation between sites. For every bromeliad, we measured a suite of environmental variables to assess the amount and quality of habitat available to the invertebrates. Then we tested for differences between sites in the bromeliad-level environmental variables. We also obtained precipitation data for every site. This file contains Table A, and Figs A-C.
(PDF)

S3 File Validation of markov network method. We validated the method by confirming it gave the same results as known interaction strengths and could predict trophic interaction strengths in simple bromeliad food webs. We took two different approaches to this confirmation. First, we ran the Markov network analysis on a three species module from Costa Rica where all interaction strengths had been established based on experiments. Second, because we have prior knowledge on the trophic ranks of every genera in the Brazilian dataset, we could test whether the Markov network method could correctly assign the trophic positions of genera. This file contains Table B and Fig D.
(PDF)

S4 File Other analyses. All of the adonis, regression and permutation output are included here. This file contains Tables C-E and Figs E-I.
(PDF)
