# Supplementary material for: A precipitation gradient drives change in macroinvertebrate composition and interactions within bromeliads
Source: PLoS One. 2018 Nov 28;13(11):e0200179. doi: 10.1371/journal.pone.0200179 (PMC6261388; doi:10.1371/journal.pone.0200179)
Supplement: S1 File — We partitioned beta diversity using Baselga’s (2009) method. In this supplementary file we show the equations used to partition beta diversity. (PDF) [file pone.0200179.s001.pdf]

S1 - Partitioning beta diversity

We used the procedure of Baselga [1], where Sørensen beta diversity for multiple-sites can be expressed as:

$$\beta_{SOR} = \frac{\left[\sum_{i<j} min(b_{ij}, b_{ji})\right] + \left[\sum_{i<j} max(b_{ij}, b_{ji})\right]}{2\left[\sum_{j} S_i - S_T\right] + \left[\sum_{i<j} min(b_{ij}, b_{ji})\right] + \left[\sum_{i<j} max(b_{ij}, b_{ji})\right]} \quad (1)$$

Where S_i is the richness in each site, b_{ij} is the number of species in site i not in site j and b_{ji} is the number of species in site j not in site i and S_T is total richness across all sites. Sørensen dissimilarity (β_{SOR}) accounts for both species turnover and nestedness. Beta diversity accounting only for pure spatial turnover is (β_{SIM}) :

$$\beta_{SIM} = \frac{\left[\sum_{i<j} min(b_{ij}, b_{ji})\right]}{2\left[\sum_{j} S_i - S_T\right] + \left[\sum_{i<j} min(b_{ij}, b_{ji})\right]} \quad (2)$$

Therefore we can use Sørensen dissimilarity (β_{SOR}) and spatial turnover (β_{SIM}) to calculate the total nestedness of species assemblages (β_{NES}):

$$\beta_{NES} = \beta_{SOR} - \beta_{SIM} \quad (3)$$

Therefore,

$$\beta_{NES} = \frac{\left[\sum_{i<j} min(b_{ij}, b_{ji})\right] + \left[\sum_{i<j} max(b_{ij}, b_{ji})\right]}{2\left[\sum_j S_i - S_T\right] + \left[\sum_{i<j} min(b_{ij}, b_{ji})\right] + \left[\sum_{i<j} max(b_{ij}, b_{ji})\right]} - \frac{\left[\sum_{i<j} min(b_{ij}, b_{ji})\right]}{2\left[\sum_j S_i - S_T\right] + \left[\sum_{i<j} min(b_{ij}, b_{ji})\right]} \quad (4)$$

References

1. Baselga A. Partitioning the turnover and nestedness components of beta diversity. Glob Ecol Biogeogr. 2009;19: 134–143.
