# Supplementary material for: A precipitation gradient drives change in macroinvertebrate composition and interactions within bromeliads
Source: PLoS One. 2018 Nov 28;13(11):e0200179. doi: 10.1371/journal.pone.0200179 (PMC6261388; doi:10.1371/journal.pone.0200179)
Supplement: S2 File — For every bromeliad, we measured a suite of environmental variables to assess the amount and quality of habitat available to the invertebrates. Then we tested for differences between sites in the bromeliad-level environmental variables. We also obtained precipitation data for every site. This file contains Table A, and Figs A-C. (PDF) [file pone.0200179.s002.pdf]

S2 - Environmental variation between sites

For every bromeliad, we measured a suite of environmental variables to assess the amount and quality of habitat available to the invertebrates: the height (cm) and diameter (cm, measured as the maximum distance between leaf tips) of the plant, maximum water volume (mL, calculated by emptying the plant and calculating how much water the plant could hold before it overflowed), actual water volume (mL), longest leaf length (cm), longest leaf width (cm), number of leaves, canopy cover (% of shaded pixels in photos taken looking directly up from the bromeliad), total detritus (g dry mass), pH, oxygen concentration (% saturation), salinity (ppt), temperature (°C), and turbidity (NTU). Water chemistry and temperature variables were measured using a portable multiparameter waterproof meter in the field as soon as the water was collected from the plant.

To test for differences between sites in environmental variables, we used a linear model and for oxygen saturation and canopy cover we used a generalized linear model with binomial family between the environmental variable and the site number.

Table A: This table shows the F value, Chisq value when using a binomial family, and the P value for comparing the environmental variables between metacommunities. All treatment degrees of freedom are 9 and residual degrees of freedom are 90.

**Environmental variable**	**F/Chisq**	**P value**
Diameter	1.501	0.159
Height	0.987	0.455
Oxygen Saturation	3.914 (Chisq)	0.916

Salinity	0.860	0.563
Canopy cover	9.084 (Chisq)	0.429
Chlorophyll	0.441	0.908
Total detritus	1.417	0.192
Turbidity	1.764	0.086
Maximum Volume	2.232	0.0267**
Actual Volume	3.854	0.0003**

We obtained precipitation information from World Clim for our sites. The finest resolution from World Clim is 30 seconds, or 1 Km^2. Sites 1-4 of our sampling are all within 1 Km^2, therefore we cannot obtain independent precipitation data from World Clim for each of these sites [1].

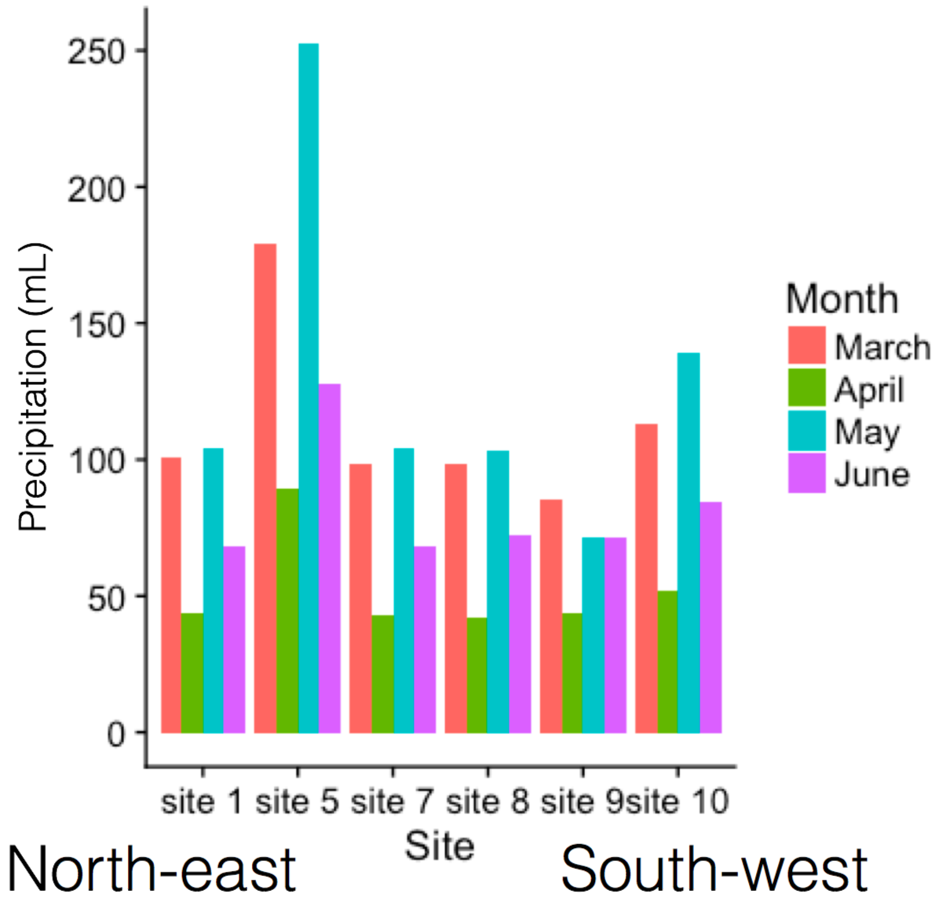

Fig A. Total precipitation (mL) for the closest 1 Km^2 of every site using WorldClim data. Sites not shown are within 1 Km^2.

We also obtained precipitation data from the year we collected from the weather stations located close to the metacommunities where we sampled [2].

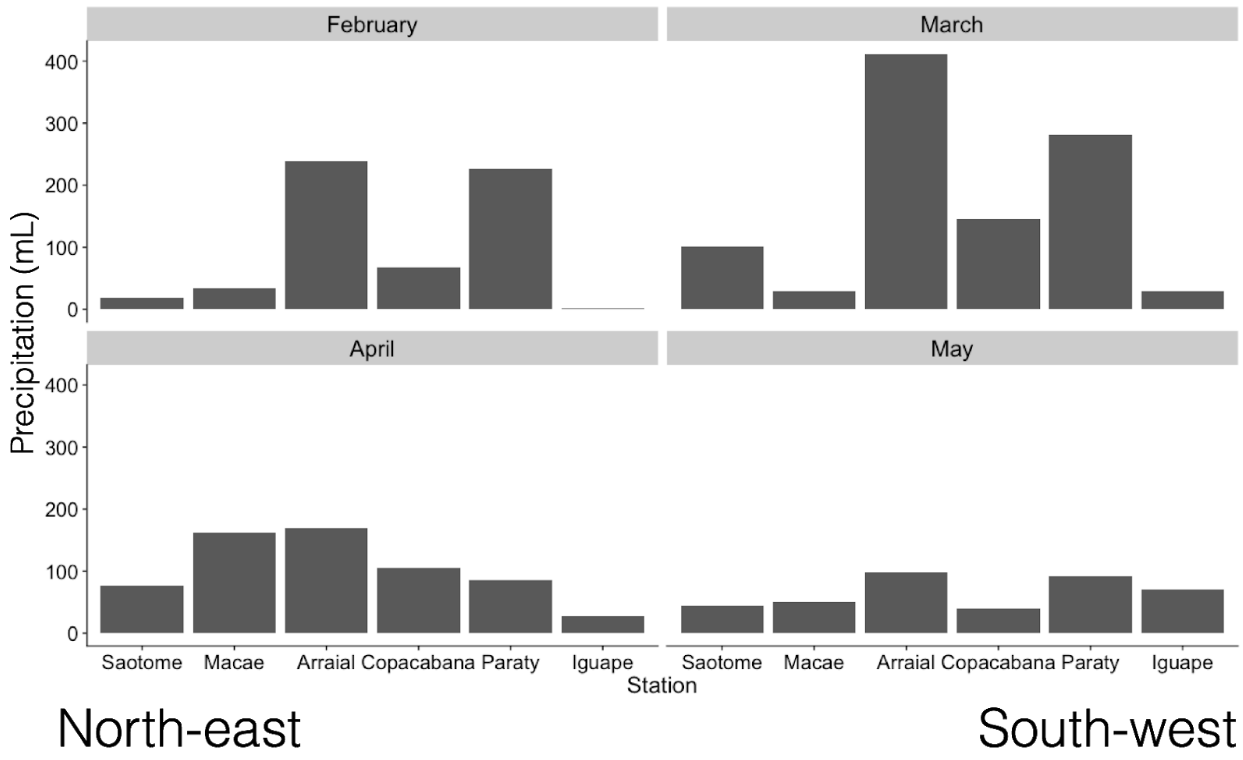

Fig B. Total precipitation (mL) for the closest weather stations to the transect. This data represents the months of sampling in 2015.

We then related the cumulative precipitation in the closest weather station to each of our sites to the actual volume of water present in the bromeliads. For each site we added the precipitation from February 1st to the date the site was sampled.

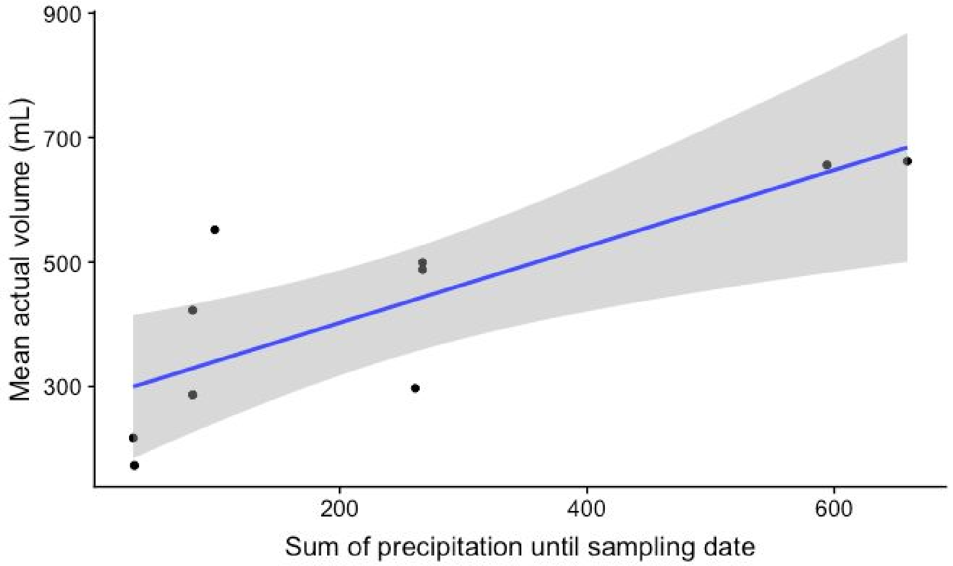

Fig C. The mean actual water volume in the bromeliads increases with the cumulative precipitation until sampling date (Intercept = 279.65, se = 53.92, slope = 0.613, se = 0.167). For each site we added the precipitation from February 1st to the date the site was sampled. We used the data from the closest weather station to the site.

References

1. Fick SE, Hijmans RJ. WorldClim 2: new 1-km spatial resolution climate surfaces for global land areas. Int J Climatol. 2017;37: 4302–4315.

2. Ministerio da agricultura P e. A. Weather stations. In: Instituto Nacional de Meterologia [Internet]. [cited 19 Dec 2016]. Available: http://www.inmet.gov.br/portal/index.php?r=home/page&page=rede_estacoes_auto_graf
