# Supplementary material for: A precipitation gradient drives change in macroinvertebrate composition and interactions within bromeliads
Source: PLoS One. 2018 Nov 28;13(11):e0200179. doi: 10.1371/journal.pone.0200179 (PMC6261388; doi:10.1371/journal.pone.0200179)
Supplement: S3 File — We validated the method by confirming it gave the same results as known interaction strengths and could predict trophic interaction strengths in simple bromeliad food webs. We took two different approaches to this confirmation. First, we ran the Markov network analysis on a three species module from Costa Rica where all interaction strengths had been established based on experiments. Second, because we have prior knowledge on the trophic ranks of every genera in the Brazilian dataset, we could test whether the Markov network method could correctly assign the trophic positions of genera. This file contains Table B and Fig D. (PDF) [file pone.0200179.s003.pdf]

S3 - Validation of markov network method

We validated the method by confirming it gave the same results as known interaction strengths and could predict trophic interaction strengths in simple bromeliad food webs. We took two different approaches to this confirmation. First, we ran the Markov network analysis on a three species module from Costa Rica where all interaction strengths had been established based on experiments [1]. Second, because we have prior knowledge on the trophic ranks of every genera in the Brazilian dataset, we could test whether the Markov network method could correctly assign the trophic positions of genera.

Markov network analysis on a known species module:

We were able to get similar interaction strengths as those expected based on direct experimentation. Using a three species module prohibits us from accounting for the indirect interactions that these three species may have with the rest of the community. However, from the experimental evidence, these three species have been shown to have strong effects on each other and we had experimental data only on this module.

We used a known species module to test the outcome of the Markov Network analyses where species interactions have been studied in detail using experiments [2]. In this species module, both the prey Culex and the predator Mecistogaster increase in abundance with bromeliad size. However, wherever the predator is present, Wyeomyia's numbers are greatly reduced. Culex is tolerant to predation due to behavioural

responses and therefore is commonly found with the predator. Using Markov Network analysis, Culex and Mecistogaster have a positive interaction strength. On the other hand, Wyeomyia and Mecistogaster have a negative interaction strength (S2 Table).

Table B: This table shows interaction strengths of the three species module studied in Hammill et al. [2].

	**Mecistogaster**	**Culex**	**Wyeomyia**
**Mecistogaster**	0.000	1.013	-2.268
**Culex**	1.013	0.000	0.936
**Wyeomyia**	-2.268	0.936	0.000

Markov networks analysis for assigning trophic rank:

For this analysis, we started by assessing the types and strengths of interactions for every combination of genera. Positive interactions mean that species are more likely to co-occur than expected by chance. Negative interactions mean that species are less likely to co-occur than expected by chance. We calculated the number of positive and negative interactions for every species, regardless of the strength across sites. We classified every species as a predator or a prey, to test if known differences in trophic positions have a different preponderance of positive (expected for prey) or negative (expected for predators) interactions. If such a pattern is found it must reflect realized trophic structure in the community since information on trophic position was not included in the estimation of interaction terms. The species or genera known to be predatory are:

the damselfly *Leptagrion andromache* nymphs, elephant mosquito larvae *Toxorynchites, Corethrella* midge larvae, horsefly larvae *Tabanidae* and cranefly larvae *Tipulidae* (Figure 1). For this analysis, we explained the total number of interactions for each genera as a function of the sign of the interaction (either positive or negative) and the trophic position. Since individual genera are present in different interactions, we included genera identity as a random effect in a generalized mixed effect model. The independent units of replication are the sites. We used a Poisson error distribution, appropriate for left-skewed count data. The interaction term of this model, between trophic position and the sign of the between-genera interaction, tests if predators and prey differ in the sign of their biological interactions.

The top predator *Leptagrion andromache* dominated negative interactions (Figure S4a), which is expected since it preys on most species in the community. In general, prey species had more positive interactions and predator species had more negative interactions compared to random expectations ($\beta = 0.564$, z value = 4.456, P value = 8.3×10^{-6}, Figure S4). This result was robust to the matrix permutations of presences (P value = 0, Appendix S4: Permutation results, S6 - S8 Fig, S5 Table). We therefore conclude that the Markov network method can accurately assess trophic interactions from observational data.

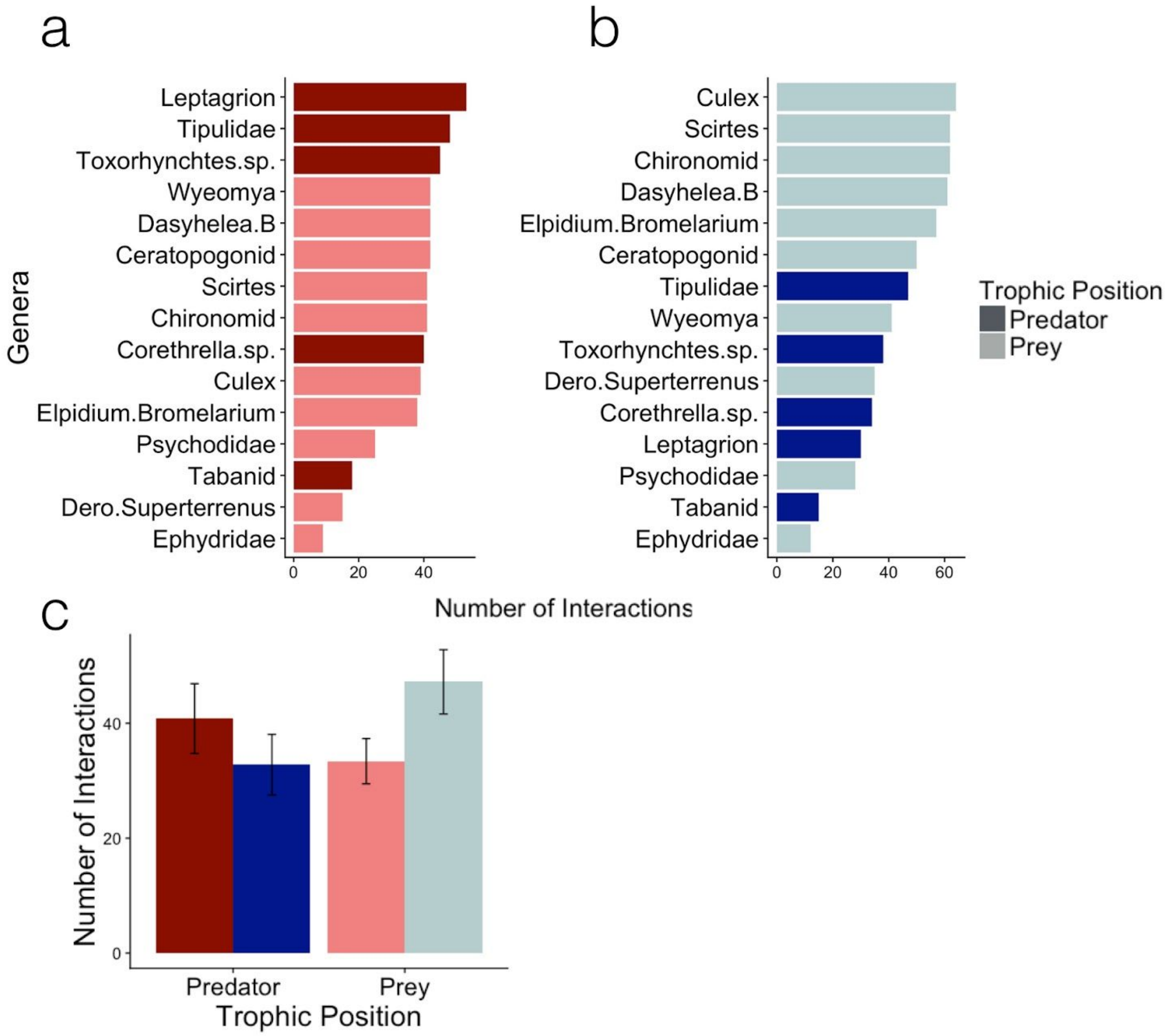

Fig D: Predators have more negative interactions and prey have more positive interactions. a) Negative interactions are dominated by *Leptagrion andromache* (top predator). b) Positive interactions are dominated by *Culex* (dipteran prey). Darker colours indicate known predatory species prior to the analysis and lighter colours indicate prey species. c) Predators have a higher number of negative interactions while prey have a higher number of positive interactions. Blue indicates positive interactions and red indicate negative interactions. Bars represent mean and standard error of the mean. Positive interactions represent species than tend to co-occur, negative interactions represent species that do not tend to co-occur.

References

1. Hammill E, Atwood TB, Srivastava DS. Predation threat alters composition and functioning of bromeliad ecosystems. Ecosystems. 2015;18: 857–866.

2. Hammill E, Atwood TB, Corvalan P, Srivastava DS. Behavioural responses to predation may explain shifts in community structure. Freshw Biol. 2014;60: 125–135.
