# Supplementary material for: A precipitation gradient drives change in macroinvertebrate composition and interactions within bromeliads
Source: PLoS One. 2018 Nov 28;13(11):e0200179. doi: 10.1371/journal.pone.0200179 (PMC6261388; doi:10.1371/journal.pone.0200179)
Supplement: S4 File — All of the adonis, regression and permutation output are included here. This file contains Tables C-E and Figs E-I. (PDF) [file pone.0200179.s004.pdf]

# Appendix S4 - Other analyses

## Pairwise multivariate analysis of variance and pairwise Tukey tests for community dispersion

Table C: This table shows the F value, $R^2$, p value and adjusted p value for pairwise multivariate analysis of variance (adonis tests) and the difference between the dispersion of communities, lower and upper boundaries of the differences and adjusted p values for pairwise tukey-tests.

| | **Pairwise multivariate analysis of variance** | | | | **Pairwise Tukey-test for community dispersion** | | | |
|---|---|---|---|---|---|---|---|---|
| **Pairs of metacommunities** | **F.Model** | **$R^2$** | **p.value** | **p.adjusted** | **Difference** | **Lower** | **Upper** | **p.adjusted** |
| 1 vs 2 | 1.634 | 0.083 | 0.205 | 1.000 | 0.043 | -0.367 | 0.454 | 1.000 |
| 1 vs 3 | 1.907 | 0.096 | 0.135 | 1.000 | 0.151 | -0.260 | 0.562 | 0.972 |
| 1 vs 4 | 1.892 | 0.095 | 0.158 | 1.000 | 0.178 | -0.232 | 0.589 | 0.922 |
| 1 vs 5 | 12.887 | 0.417 | 0.001 | 0.045* | -0.159 | -0.569 | 0.252 | 0.961 |
| 1 vs 6 | 9.186 | 0.338 | 0.001 | 0.045* | 0.195 | -0.216 | 0.605 | 0.873 |
| 1 vs 7 | 4.272 | 0.192 | 0.010 | 0.450 | 0.103 | -0.308 | 0.513 | 0.998 |
| 1 vs 8 | 15.549 | 0.463 | 0.001 | 0.045* | -0.163 | -0.573 | 0.248 | 0.955 |
| 1 vs 9 | 6.718 | 0.272 | 0.001 | 0.045* | 0.062 | -0.348 | 0.473 | 1.000 |
| 1 vs 10 | 11.157 | 0.383 | 0.001 | 0.045* | 0.036 | -0.374 | 0.447 | 1.000 |

| | | | | | | | | |
|---|---|---|---|---|---|---|---|---|
| 2 vs 3 | 0.743 | 0.040 | 0.614 | 1.000 | 0.108 | -0.303 | 0.518 | 0.997 |
| 2 vs 4 | 2.355 | 0.116 | 0.052 | 1.000 | 0.135 | -0.276 | 0.545 | 0.987 |
| 2 vs 5 | 13.273 | 0.424 | 0.001 | 0.045* | -0.202 | -0.613 | 0.208 | 0.846 |
| 2 vs 6 | 7.746 | 0.301 | 0.001 | 0.045* | 0.151 | -0.259 | 0.562 | 0.971 |
| 2 vs 7 | 3.849 | 0.176 | 0.007 | 0.315 | 0.059 | -0.351 | 0.470 | 1.000 |
| 2 vs 8 | 15.564 | 0.464 | 0.001 | 0.045* | -0.206 | -0.617 | 0.205 | 0.831 |
| 2 vs 9 | 7.690 | 0.299 | 0.001 | 0.045* | 0.019 | -0.392 | 0.429 | 1.000 |
| 2 vs 10 | 7.988 | 0.307 | 0.002 | 0.090 | -0.007 | -0.418 | 0.403 | 1.000 |
| 3 vs 4 | 1.717 | 0.087 | 0.165 | 1.000 | 0.027 | -0.383 | 0.438 | 1.000 |
| 3 vs 5 | 8.485 | 0.320 | 0.001 | 0.045* | -0.310 | -0.720 | 0.101 | 0.311 |
| 3 vs 6 | 4.413 | 0.197 | 0.003 | 0.135 | 0.044 | -0.367 | 0.454 | 1.000 |
| 3 vs 7 | 4.304 | 0.193 | 0.002 | 0.090 | -0.048 | -0.459 | 0.362 | 1.000 |
| 3 vs 8 | 13.604 | 0.430 | 0.001 | 0.045* | -0.314 | -0.724 | 0.097 | 0.294 |
| 3 vs 9 | 4.701 | 0.207 | 0.001 | 0.045* | -0.089 | -0.500 | 0.322 | 0.999 |
| 3 vs 10 | 6.041 | 0.251 | 0.001 | 0.045* | -0.115 | -0.525 | 0.296 | 0.996 |
| 4 vs 5 | 5.571 | 0.236 | 0.001 | 0.045* | -0.337 | -0.747 | 0.074 | 0.205 |
| 4 vs 6 | 6.196 | 0.256 | 0.001 | 0.045* | 0.016 | -0.394 | 0.427 | 1.000 |
| 4 vs 7 | 3.214 | 0.152 | 0.010 | 0.450 | -0.075 | -0.486 | 0.335 | 1.000 |

| | | | | | | | | |
|---|---|---|---|---|---|---|---|---|
| 4 vs 8 | 8.524 | 0.321 | 0.001 | 0.045* | -0.341 | -0.751 | 0.070 | 0.193 |
| 4 vs 9 | 7.137 | 0.284 | 0.001 | 0.045* | -0.116 | -0.527 | 0.295 | 0.996 |
| 4 vs 10 | 10.432 | 0.367 | 0.001 | 0.045* | -0.142 | -0.553 | 0.269 | 0.981 |
| 5 vs 6 | 2.857 | 0.137 | 0.021 | 0.945 | 0.353 | -0.057 | 0.764 | 0.155 |
| 5 vs 7 | 6.691 | 0.271 | 0.001 | 0.045* | 0.261 | -0.149 | 0.672 | 0.556 |
| 5 vs 8 | 4.122 | 0.186 | 0.007 | 0.315 | -0.004 | -0.414 | 0.407 | 1.000 |
| 5 vs 9 | 6.496 | 0.265 | 0.001 | 0.045* | 0.221 | -0.190 | 0.631 | 0.767 |
| 5 vs 10 | 12.187 | 0.404 | 0.001 | 0.045* | 0.195 | -0.216 | 0.605 | 0.872 |
| 6 vs 7 | 5.470 | 0.233 | 0.001 | 0.045* | -0.092 | -0.503 | 0.319 | 0.999 |
| 6 vs 8 | 6.412 | 0.263 | 0.001 | 0.045* | -0.357 | -0.768 | 0.053 | 0.145 |
| 6 vs 9 | 3.823 | 0.175 | 0.004 | 0.180 | -0.132 | -0.543 | 0.278 | 0.988 |
| 6 vs 10 | 7.344 | 0.290 | 0.001 | 0.045* | -0.158 | -0.569 | 0.252 | 0.961 |
| 7 vs 8 | 10.299 | 0.364 | 0.001 | 0.045* | -0.265 | -0.676 | 0.145 | 0.535 |
| 7 vs 9 | 7.227 | 0.286 | 0.001 | 0.045* | -0.041 | -0.451 | 0.370 | 1.000 |
| 7 vs 10 | 10.481 | 0.368 | 0.001 | 0.045* | -0.066 | -0.477 | 0.344 | 1.000 |
| 8 vs 9 | 8.375 | 0.318 | 0.001 | 0.045* | 0.225 | -0.186 | 0.635 | 0.748 |
| 8 vs 10 | 16.800 | 0.483 | 0.001 | 0.045* | 0.199 | -0.212 | 0.609 | 0.858 |
| 9 vs 10 | 2.282 | 0.113 | 0.073 | 1.000 | -0.026 | -0.437 | 0.385 | 1.000 |

## Regression results

This graph shows the positive and negative mean regressions for every species.

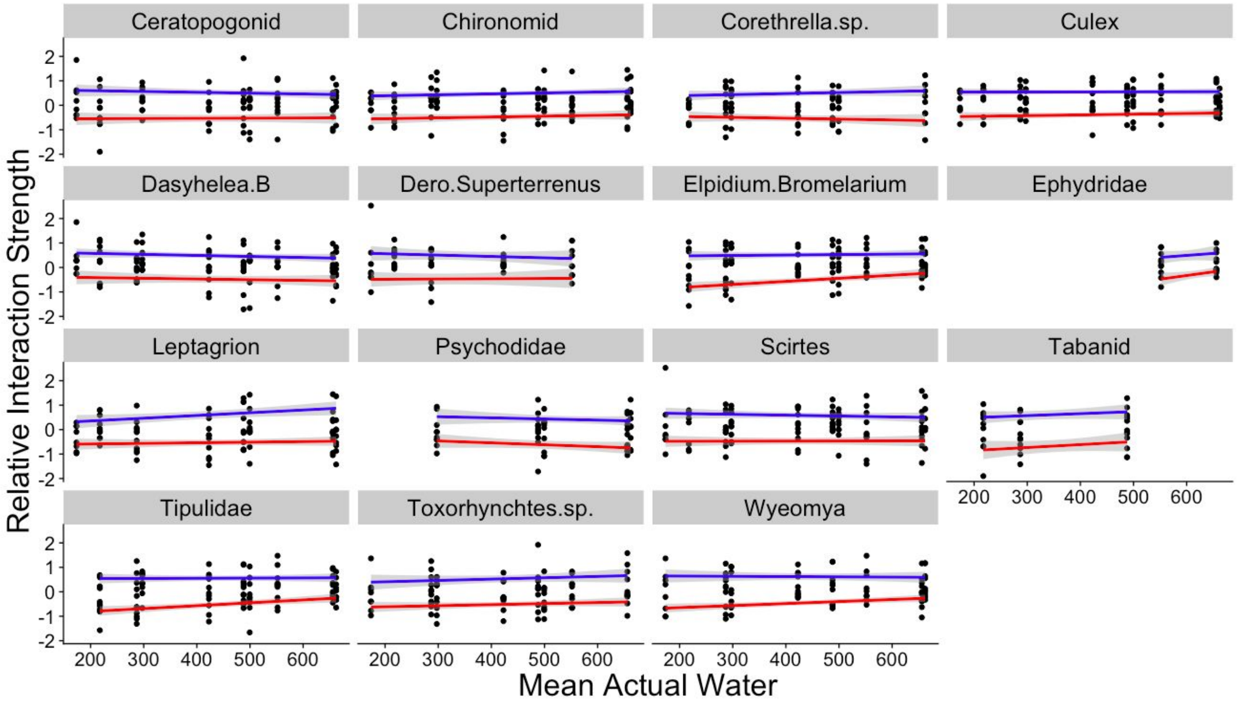


Fig E: Relative interaction strength against mean bromeliad water volume. Every panel represents a different focal species. Blue regression lines represent the positive interactions and the red regression lines represent the negative interactions.

Table D: This table shows the slope and the p value (corrected and uncorrected for multiple tests) for the linear regression between positive or negative relative interaction strengths and mean bromeliad volume.

| **Species** | **Negative slope** | **P value** | **Corrected for multiple tests P value** | **Positive slope** | **P value** | **Corrected for multiple tests P value** |
|---|---|---|---|---|---|---|
| Tipulidae | 1.179e-03 | 0.0012** | 0.0178** | 7.12e-05 | 0.838 | 1.00 |

| | | | | | | |
|---|---|---|---|---|---|---|
| Corethrella.sp. | -3.58e-04 | 0.435 | 1.00 | 4.25e-04 | 0.291 | 1.00 |
| Leptagrion | 2.44e-04 | 0.488 | 1.00 | 1.116e-03 | 0.020** | 0.312 |
| Elpidium | 1.251e-03 | 0.001** | 0.0163** | 1.82e-04 | 0.609 | 1.00 |
| Dero | 9.69e-05 | 0.885 | 1.00 | -5.53e-04 | 0.418 | 1.00 |
| Chironomid | 3.42e-04 | 0.337 | 1.00 | 3.73e-04 | 0.209 | 1.00 |
| Scirtes | 2.97e-05 | 0.938 | 1.00 | -3.47e-04 | 0.34 | 1.00 |
| Ceratopogonid | 6.69e-05 | 0.872 | 1.00 | -3.43e-04 | 0.34 | 1.00 |
| Culex | 2.96e-04 | 0.295 | 1.00 | 3.27e-05 | 0.891 | 1.00 |
| Tabanid | 1.19e-03 | 0.256 | 1.00 | 8.08e-04 | 0.277 | 1.00 |
| Toxorhynchtes | 4.3e-04 | 0.216 | 1.00 | 5.49e-04 | 0.300 | 1.00 |
| Wyeomya | 8.24e-04 | 0.004** | 0.06** | -1.08e-04 | 0.794 | 1.00 |
| Psychodidae | -7.78e-04 | 0.175 | 1.00 | -4.67e-04 | 0.428 | 1.00 |
| Ephydridae | 3.00e-03 | 0.057 | 0.690 | 1.66e-03 | 0.38 | 1.00 |
| Dasyhelea.B | -2.91e-04 | 0.513 | 1.00 | -4.25e-04 | 0.180 | 1.00 |

## Permutation results

Since we only obtain one set of interaction strength values for each site, and we wanted to ensure that the results we obtained were not due to a random combination of presence and absences, we permuted the presence-absence matrix for each site, and

then re-calculated the interaction strengths. We permuted the presence of the species by keeping the number of species in each bromeliad constant across each permutation. For each of our metacommunities, we created 10,000 reshuffled metacommunities. After calculating the interaction strength for each site, we recalculated 1) the effect of the type of sign and trophic position on the number of interactions and 2) the effect of water volume on the positive or negative interaction strength of the species.

The effect of the type of sign and trophic position on the number of interactions:

To assess the effect of type of sign of interaction and trophic position on the number of interactions we ran a generalized linear model. For this model, we used the number of interactions each species had for the full data set as a function of the the sign of the species interaction (either positive or negative) and the trophic position using the species identity as a random effect; the units of replication are the metacommunities. The interaction term of this model, between sign of species interaction and trophic position, tests if predators and prey have different distributions of the sign of their interactions. Here we show the distribution of the interaction term of the model from all the permutations vs the interaction term we obtained from our data.

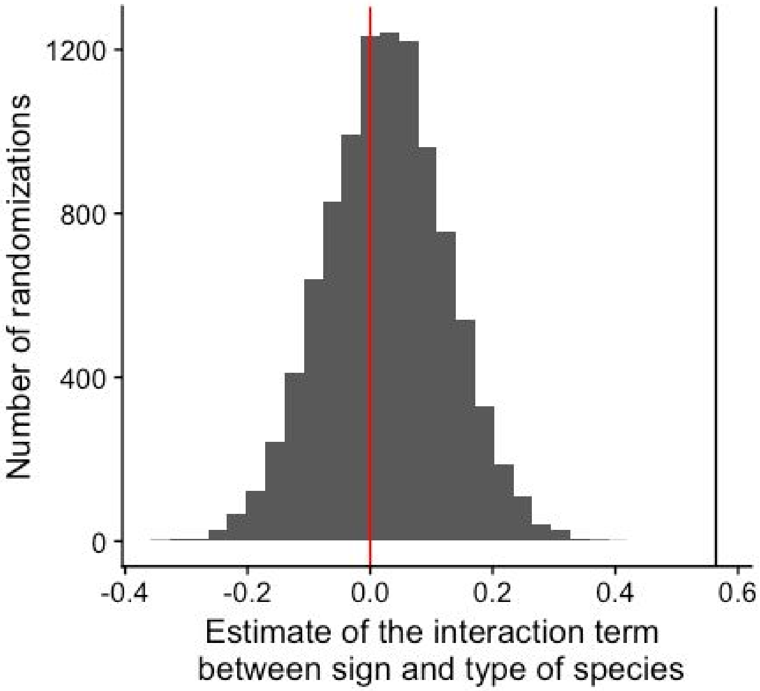


Fig F: Distribution of interaction terms after the presence of species was permuted 10,000 times. Red line represents 0 and the black line represents the actual interaction term found in our original data.

Here we see that the interaction term of the model we obtained from our data does not overlap the distribution.

We are calculating the P value for this permutation test by the proportion of values from the distribution that are greater than the value we obtained from the original data. Here the p-value is 0.

**The effect of water volume on the positive or negative interaction strength of the species:**

To assess the effect of water volume on the positive or negative interaction strength of the species we used a linear regression. Here we compare the slope of that regression for each species vs the distribution of slopes obtained from the permutation of the presence matrix.

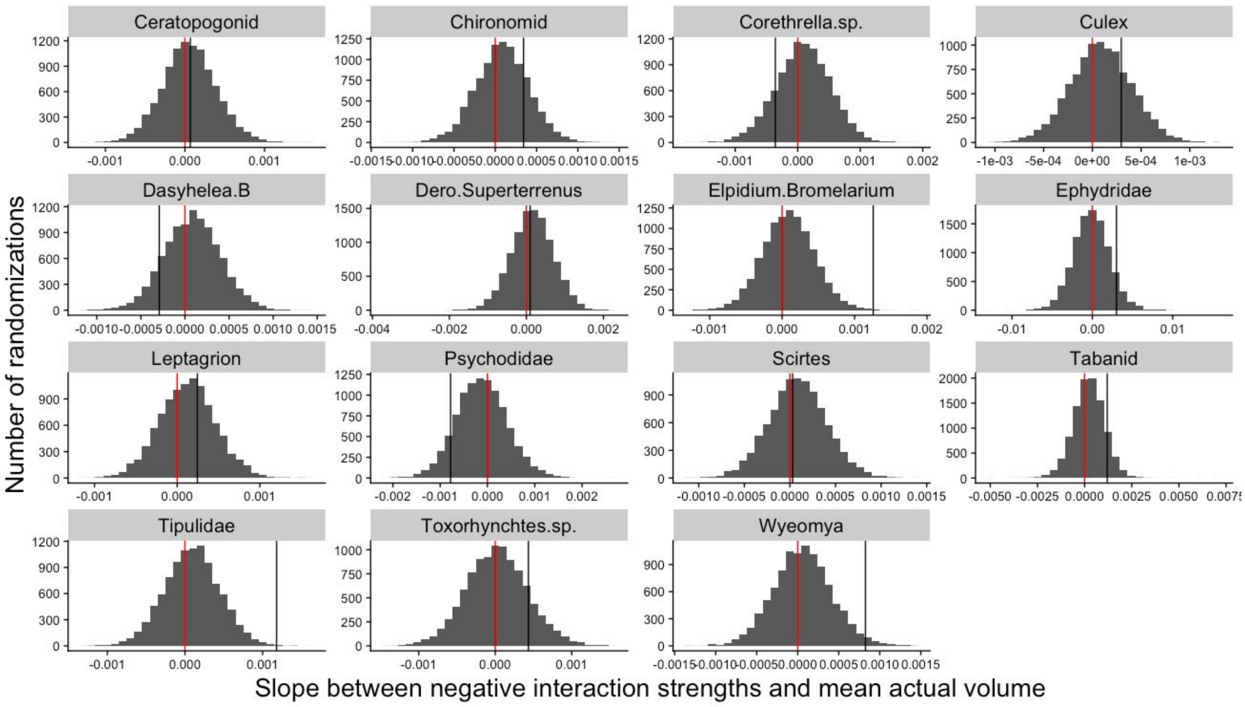


Fig G: Distribution of negative slopes after the presence of species was permuted 10,000 times. Red line represents 0 and the black line represents the actual slope found in our original data.

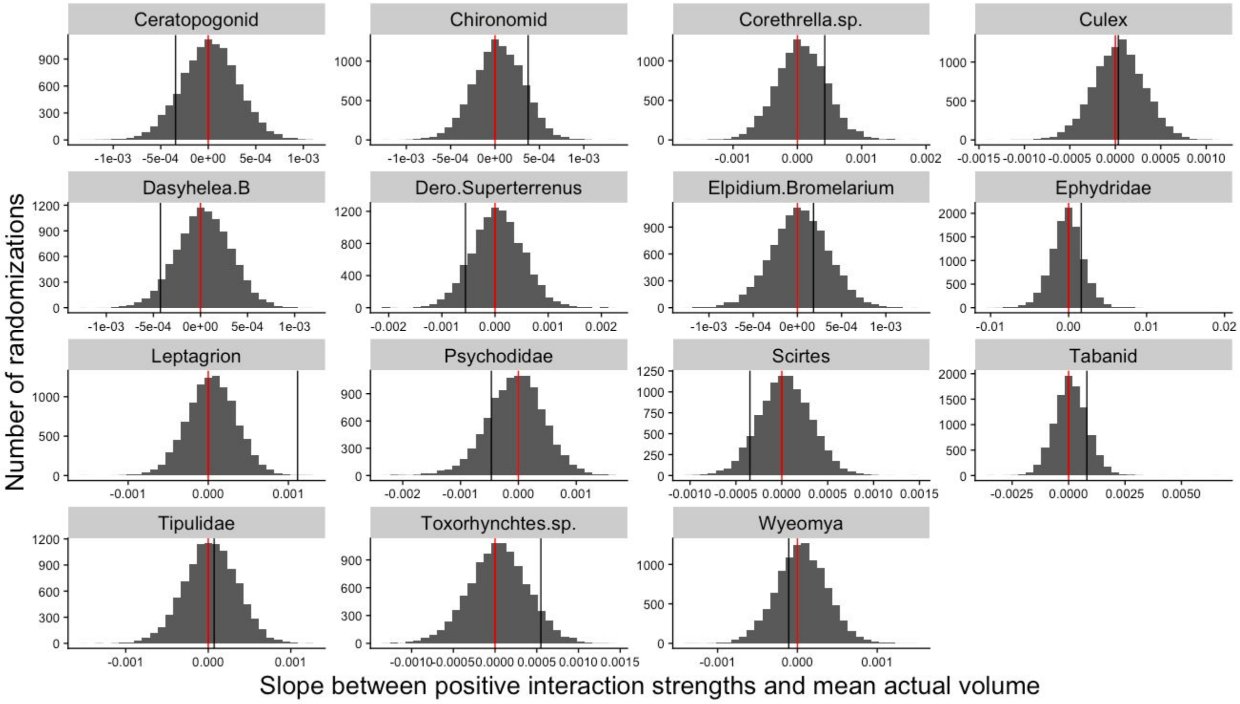


Fig H: Distribution of positive slopes after the presence of species was permuted 10,000 times. Red line represents 0 and the black line represents the actual slope found in our original data.

We are calculating the P value for this permutation test by the proportion of values from the distribution that are greater than the value we obtained from the original data.

Table E: This table shows the p value for the permutation test for linear regression between positive or negative relative interaction strengths and mean bromeliad volume.

| **Species** | **P value for positive interaction values** | **P value for negative interaction values** |
|---|---|---|
| Tipulidae | 0.448 | 0.001 ** |
| Corethrella.sp. | 0.175 | 0.845 |
| Leptagrion | 0.0005 ** | 0.360 |
| Elpidium bromelarium | 0.320 | 0.001 ** |

| | | |
|---|---|---|
| Dero superterrenus | 0.897 | 0.545 |
| Scirtes | 0.911 | 0.569 |
| Chironomid | 0.118 | 0.209 |
| Ceratopogonid | 0.886 | 0.481 |
| Culex | 0.513 | 0.252 |
| Tabanid | 0.165 | 0.115 |
| Toxorhynchtes.sp. | 0.077 | 0.149 |
| Wyeomya | 0.698 | 0.019 ** |
| Psychodidae | 0.812 | 0.892 |
| Ephydridae | 0.169 | 0.080 |
| Dasyhelea.B | 0.947 | 0.881 |

## Relative interaction strength vs presence

The negative interaction strength between two species can be due to the absence of one caused by the local community environment (for example, low water volume filters out certain species). Therefore, we wanted to test if the slope between the presence of any given species and water volume in a bromeliad was related to their interaction strength to other species. The expectation here was that if the interaction strength at the site scale is driven by the filtering of the local environment, then they would be more

likely to engage in negative interactions if the slope between presence and water volume is positive. Here we plotted the slope of the relationship between the presence of each species and water volume in the bromeliad, against the interaction strength value that they have against the tipulid on that site. We find that there is no relationship between this slope and the interaction strength with the Tipulid.

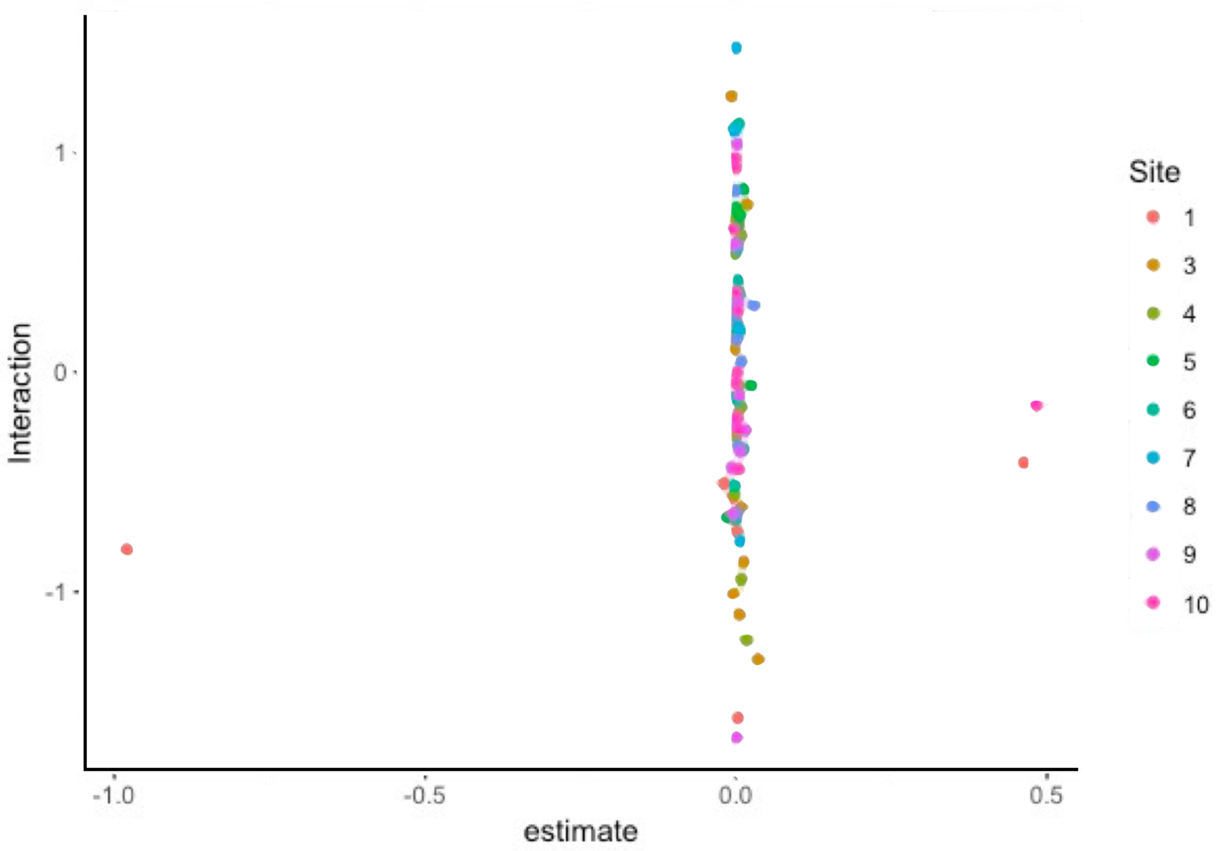


Fig I: The estimate of a logistic regression between actual water volume in the bromeliad and the presence of the species versus the relative interaction strength with the tipulid at each site.
